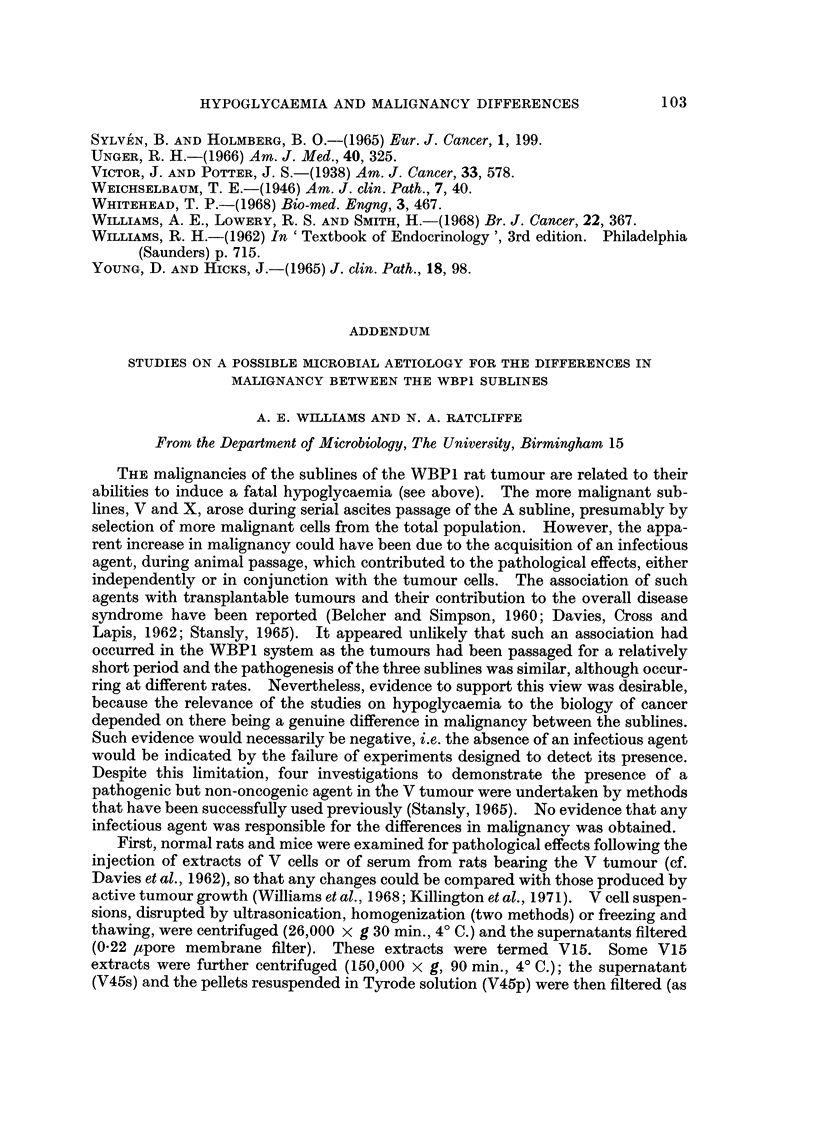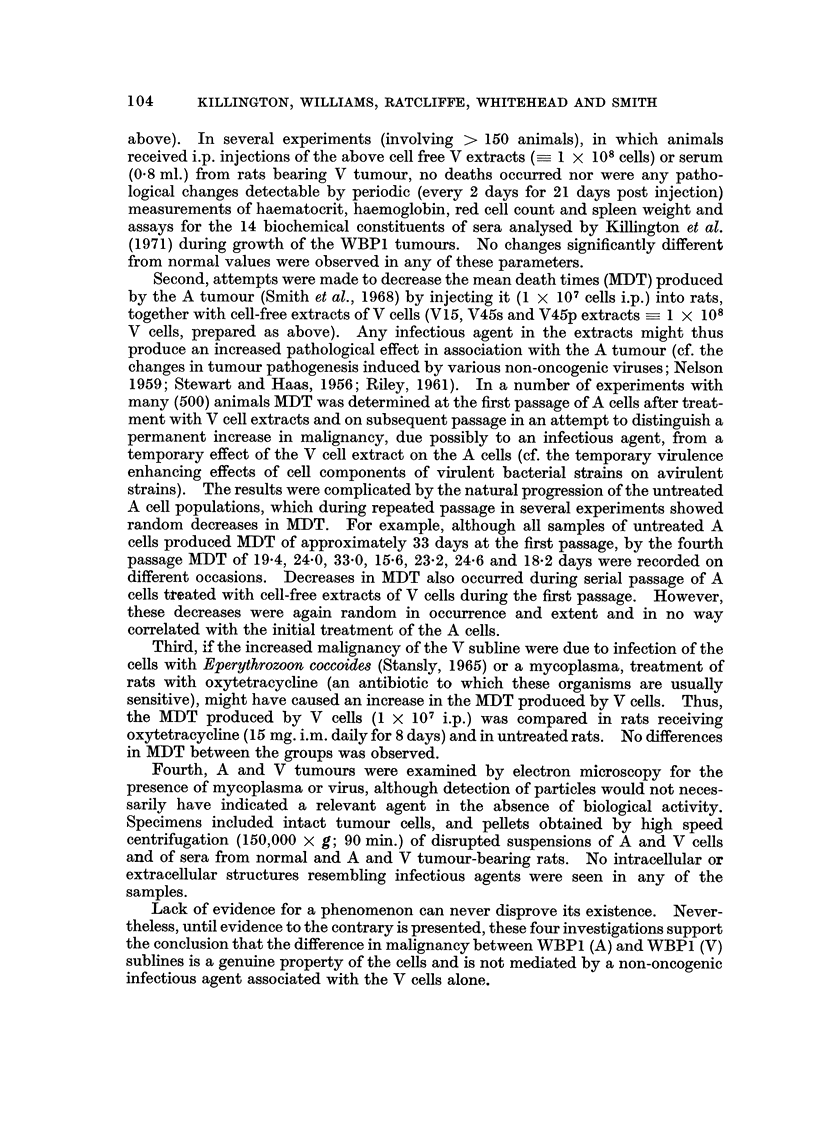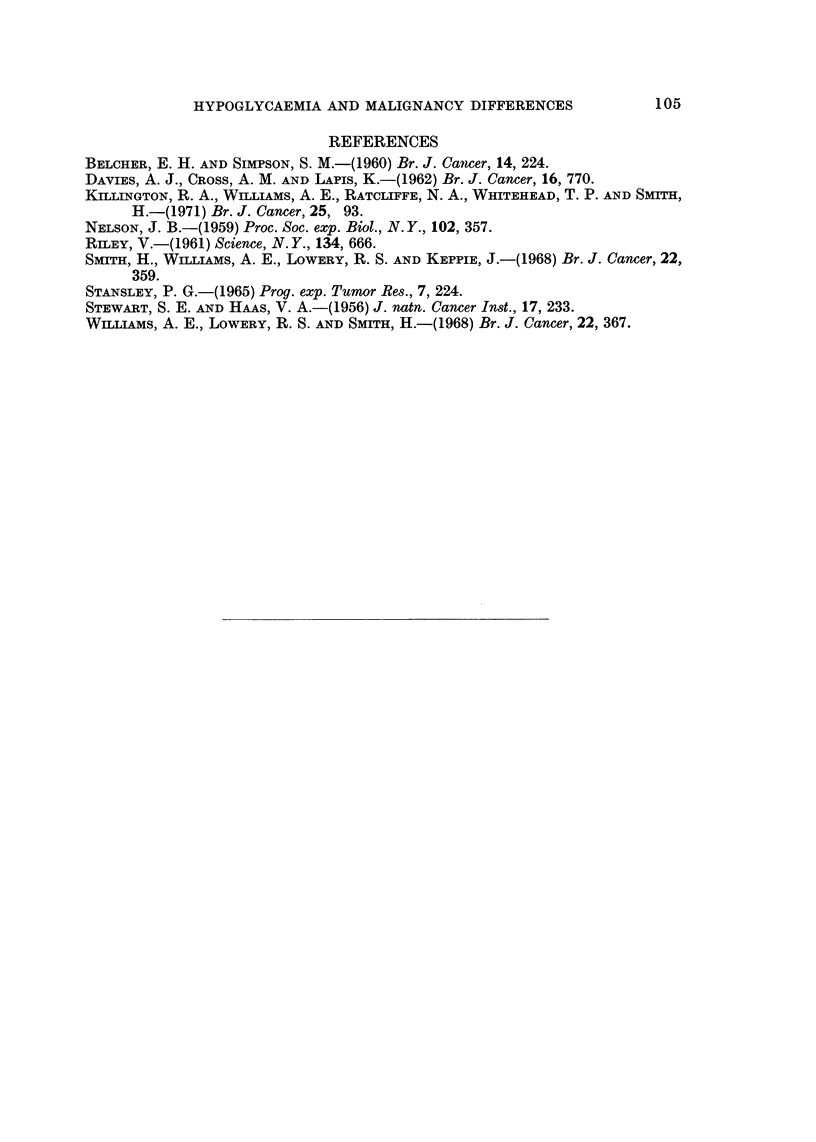# Studies on a possible microbial aetiology for the differences in malignancy between the WBP1 sublines.

**DOI:** 10.1038/bjc.1971.14

**Published:** 1971-03

**Authors:** A. E. Williams, N. A. Ratcliffe


Williams, A. E., Lowery, R. S. and Smith, H.—(1968) *Br. J. Cancer*, **22**, 367.

Williams, R. H.—(1962) *In* ' Textbook of Endocrinology ', 3rd edition. Philadelphia (Saunders) p. 715.

Young, D. and Hicks, J.—(1965) *J. clin. Path.*, **18**, 98.

### ADDENDUM

### STUDIES ON A POSSIBLE MICROBIAL AETIOLOGY FOR THE DIFFERENCES IN MALIGNANCY BETWEEN THE WBP1 SUBLINES

A. E. WILLIAMS AND N. A. RATCLIFFE

*From the Department of Microbiology, The University, Birmingham* 15

The malignancies of the sublines of the WBP1 rat tumour are related to their abilities to induce a fatal hypoglycaemia (see above). The more malignant sublines, V and X, arose during serial ascites passage of the A subline, presumably by selection of more malignant cells from the total population. However, the apparent increase in malignancy could have been due to the acquisition of an infectious agent, during animal passage, which contributed to the pathological effects, either independently or in conjunction with the tumour cells. The association of such agents with transplantable tumours and their contribution to the overall disease syndrome have been reported (Belcher and Simpson, 1960; Davies, Cross and Lapis, 1962; Stansly, 1965). It appeared unlikely that such an association had occurred in the WBP1 system as the tumours had been passaged for a relatively short period and the pathogenesis of the three sublines was similar, although occurring at different rates. Nevertheless, evidence to support this view was desirable, because the relevance of the studies on hypoglycaemia to the biology of cancer depended on there being a genuine difference in malignancy between the sublines. Such evidence would necessarily be negative, *i.e.* the absence of an infectious agent would be indicated by the failure of experiments designed to detect its presence. Despite this limitation, four investigations to demonstrate the presence of a pathogenic but non-oncogenic agent in the V tumour were undertaken by methods that have been successfully used previously (Stansly, 1965). No evidence that any infectious agent was responsible for the differences in malignancy was obtained.

First, normal rats and mice were examined for pathological effects following the injection of extracts of V cells or of serum from rats bearing the V tumour (cf. Davies *et al.*, 1962), so that any changes could be compared with those produced by active tumour growth (Williams *et al.*, 1968; Killington *et al.*, 1971). V cell suspensions, disrupted by ultrasonication, homogenization (two methods) or freezing and thawing, were centrifuged (26,000 $\times$ $g$ 30 min., 4° C.) and the supernatants filtered (0·22 $\mu$pore membrane filter). These extracts were termed V15. Some V15 extracts were further centrifuged (150,000 $\times$ $g$, 90 min., 4° C.); the supernatant (V45s) and the pellets resuspended in Tyrode solution (V45p) were then filtered (as above). In several experiments (involving $> 150$ animals), in which animals received i.p. injections of the above cell free V extracts ($\equiv 1 \times 10^8$ cells) or serum (0·8 ml.) from rats bearing V tumour, no deaths occurred nor were any pathological changes detectable by periodic (every 2 days for 21 days post injection) measurements of haematocrit, haemoglobin, red cell count and spleen weight and assays for the 14 biochemical constituents of sera analysed by Killington *et al.* (1971) during growth of the WBP1 tumours. No changes significantly different from normal values were observed in any of these parameters.

Second, attempts were made to decrease the mean death times (MDT) produced by the A tumour (Smith *et al.*, 1968) by injecting it ($1 \times 10^7$ cells i.p.) into rats, together with cell-free extracts of V cells (V15, V45s and V45p extracts $\equiv 1 \times 10^8$ V cells, prepared as above). Any infectious agent in the extracts might thus produce an increased pathological effect in association with the A tumour (cf. the changes in tumour pathogenesis induced by various non-oncogenic viruses; Nelson 1959; Stewart and Haas, 1956; Riley, 1961). In a number of experiments with many (500) animals MDT was determined at the first passage of A cells after treatment with V cell extracts and on subsequent passage in an attempt to distinguish a permanent increase in malignancy, due possibly to an infectious agent, from a temporary effect of the V cell extract on the A cells (cf. the temporary virulence enhancing effects of cell components of virulent bacterial strains on avirulent strains). The results were complicated by the natural progression of the untreated A cell populations, which during repeated passage in several experiments showed random decreases in MDT. For example, although all samples of untreated A cells produced MDT of approximately 33 days at the first passage, by the fourth passage MDT of 19·4, 24·0, 33·0, 15·6, 23·2, 24·6 and 18·2 days were recorded on different occasions. Decreases in MDT also occurred during serial passage of A cells treated with cell-free extracts of V cells during the first passage. However, these decreases were again random in occurrence and extent and in no way correlated with the initial treatment of the A cells.

Third, if the increased malignancy of the V subline were due to infection of the cells with *Eperythrozoon coccoides* (Stansly, 1965) or a mycoplasma, treatment of rats with oxytetracycline (an antibiotic to which these organisms are usually sensitive), might have caused an increase in the MDT produced by V cells. Thus, the MDT produced by V cells ($1 \times 10^7$ i.p.) was compared in rats receiving oxytetracycline (15 mg. i.m. daily for 8 days) and in untreated rats. No differences in MDT between the groups was observed.

Fourth, A and V tumours were examined by electron microscopy for the presence of mycoplasma or virus, although detection of particles would not necessarily have indicated a relevant agent in the absence of biological activity. Specimens included intact tumour cells, and pellets obtained by high speed centrifugation ($150{,}000 \times g$; 90 min.) of disrupted suspensions of A and V cells and of sera from normal and A and V tumour-bearing rats. No intracellular or extracellular structures resembling infectious agents were seen in any of the samples.

Lack of evidence for a phenomenon can never disprove its existence. Nevertheless, until evidence to the contrary is presented, these four investigations support the conclusion that the difference in malignancy between WBP1 (A) and WBP1 (V) sublines is a genuine property of the cells and is not mediated by a non-oncogenic infectious agent associated with the V cells alone.

### REFERENCES

BELCHER, E. H. AND SIMPSON, S. M.—(1960) *Br. J. Cancer*, **14**, 224.

DAVIES, A. J., CROSS, A. M. AND LAPIS, K.—(1962) *Br. J. Cancer*, **16**, 770.

KILLINGTON, R. A., WILLIAMS, A. E., RATCLIFFE, N. A., WHITEHEAD, T. P. AND SMITH, H.—(1971) *Br. J. Cancer*, **25**, 93.

NELSON, J. B.—(1959) *Proc. Soc. exp. Biol., N.Y.*, **102**, 357.

RILEY, V.—(1961) *Science, N.Y.*, **134**, 666.

SMITH, H., WILLIAMS, A. E., LOWERY, R. S. AND KEPPIE, J.—(1968) *Br. J. Cancer*, **22**, 359.

STANSLEY, P. G.—(1965) *Prog. exp. Tumor Res.*, **7**, 224.

STEWART, S. E. AND HAAS, V. A.—(1956) *J. natn. Cancer Inst.*, **17**, 233.

WILLIAMS, A. E., LOWERY, R. S. AND SMITH, H.—(1968) *Br. J. Cancer*, **22**, 367.